# Microwave Assisted Sol-Gel Synthesis of Silica-Spider Silk Composites

**DOI:** 10.3390/molecules24142521

**Published:** 2019-07-10

**Authors:** Abul Bashar Mohammad Giasuddin, David W. Britt

**Affiliations:** Department of Biological Engineering, Utah State University, Logan, UT 84322-4105, USA

**Keywords:** microwave, spider silk, silica, ORMOSIL, inorganic-organic composites

## Abstract

This study introduces a simple and environmentally friendly method to synthesize silica-protein nanocomposite materials using microwave energy to solubilize hydrophobic protein in an aqueous solution of pre-hydrolyzed organo- or fluoro-silane. Sol-gel functionality can be enhanced through biomacromolecule incorporation to tune mechanical properties, surface energy, and biocompatibility. Here, synthetic spider silk protein and organo- and fluoro-silane precursors were dissolved and mixed in weakly acidic aqueous solution using microwave technology. Scanning electron microscopy (SEM) and Atomic force microscopy (AFM) images revealed the formation of spherical nanoparticles with sizes ranging from 100 to 500 nm depending, in part, on silane fluoro- or organo-side chain chemistry. The silane-protein interaction in the nanocomposite was assessed through infrared spectroscopy. Deconvoluted ATR-FTIR (Attenuated total reflectance Fourier-transform infrared spectroscopy) spectra revealed silane chemistry-specific conformational changes in the protein-silane nanocomposites. Relative to microwave-solubilized spider silk protein, the β structure content increased by 14% in the spider silk-organo-silica nanocomposites, but decreased by a net 20% in the spider silk-fluoro-silica nanocomposites. Methods of tuning the secondary structures, and in particular β-sheets that are the cross-linking moieties in spider silks and other self-assembling fibrillar proteins, may provide a unique means to promote protein interactions, favor subsequent epitaxial growth process, and enhance the properties of the protein-silane nanocomposites.

## 1. Introduction

Silica particles are widely used in industry, medicine, and nanotechnology [1,2]. Bulk and surface properties are modified through the incorporation of small molecules such as surfactants to introduce porosity during synthesis, or coating with organofunctional silanes post-synthesis to impart defined surface chemistries [3]. If the silane function group is an alkyl- or aryl-moiety the resulting particle or gel is termed an organically modified silica (ORMOSIL). Organic modifications are often incorporated as a functional bridge to promote adhesion with additional molecules. Biomacromolecules bonding to silica particles, surfaces, and within porous gels can be improved through ORMOSIL selection [4,5]. Preserving immobilized biomolecule function, through defined orientation and retention of native confirmations on/in silicas is critical for biomedical and diagnostics applications [6]. In particular, protein-silica composite materials are explored for application in biomedical fields such as immunology, cancer research, and drug delivery [7,8,9,10]. These hybrid materials are also investigated in materials science technologies such as self-assembled materials, quantum dot bioconjugates, sensors, and inorganic materials synthesis [11,12,13,14,15,16,17].

Silica-based biomaterials have potential applications in tissue engineering, where biomineralization is necessary for bone and tooth repair, while simultaneously serving as a drug delivery system to stimulate surrounding tissues or prevent infection. Structural integrity can be enhanced through incorporation of fibrillar protein assemblies such as those observed for spider silk. Recombinant spider silk (SS) protein is a promising biomaterial with huge potential in the textiles, biomedical, and manufacturing industry in the form of fibers, films, hydrogels, lyogels, and adhesives [18,19,20,21,22,23,24]. SS based materials have shown potential in tissue engineering through surface modifications that promote fibroblast cell attachment and proliferation [25,26,27,28,29,30]. The extraordinary mechanical strength of SS comes from its β-sheet dominated secondary structures that form the basis for self-assembly through strong physical interactions [14]. However, SS proteins are insoluble in aqueous solution, and conventionally dissolved in 1,1,1,3,3,3-hexafluoro-2-propanol (HFIP) to generate spin dopes to create fibers, films, gels, and foams [31,32,33,34]. HFIP, however, is expensive and poses significant health and environmental risks. Recently, a simple microwave method has been introduced to dissolve SS in aqueous solution, providing a cost-effective and green approach to preparing SS-functionalized materials and surfaces [35].

Here, we investigate addition of simple organo- and fluoro-silanes (ORMOSILs and F-ORMOSILs) to SS as a means to tune properties of the resulting sol-gel inorganic/organic composite. Alkoxysilanes are often used as the precursor for silicone, a widely used adhesive to bridge inorganic materials with organic molecules [36]. Hydrolyzed ORMOSILs and F-ORMOSILS exhibit a strong ability to induce secondary structures of globular proteins [37]. We select hydrophobic n-propyltrimethoxy silane (nPM) and 3,3,3-trifluoropropyl trimethoxy silane (3F) as both have been shown to influence the molten globule transition and secondary structures of beta-lactoglobulin and albumin [37], which are low MW, water soluble proteins in contrast to SS. We hypothesize that hydrolyzed nPM and 3F can induce secondary structure in SS and upon condensation form bio-inorganic nanocomposites consisting of SS and silica. Using microwave-assisted dissolution of SS in the presence of hydrolyzed silanes followed by base-initiated condensation yields hybrid spider silk-silica nanocomposites under purely aqueous conditions. Spherical sub-micron particles are observed for both 3F and nPM, however, the two silanes distinctly influence the SS secondary structures as determined by FTIR analysis. The β structural content increased by 10% in the SS-nPM nanocomposites, but decreased by a net 28% in the SS-3F nanocomposites. The ability to tune protein secondary structures in the protein-ORMOSIL composite may provide a means to control subsequent growth processes, such as biomineralization with which biological function and mechanical properties of these composite organic/inorganic biomaterials can be modified.

## 2. Results and Discussion

**Figure 1:**
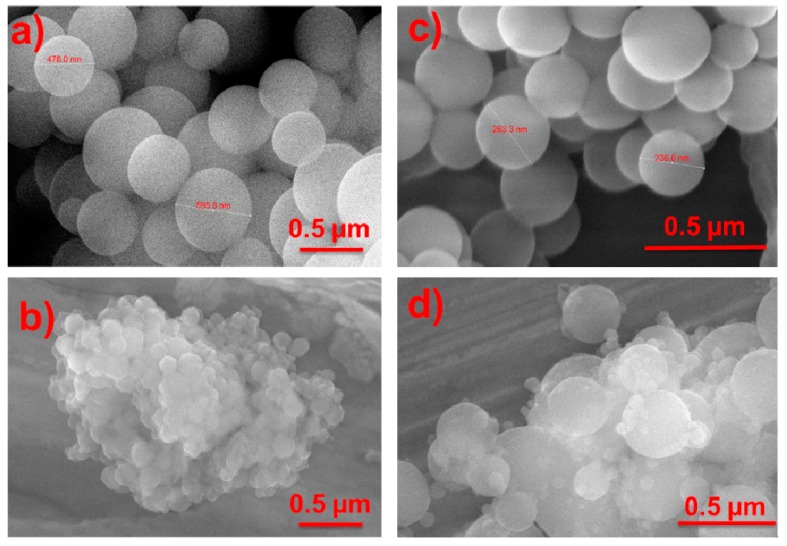
SEM images of (**a**) nPM based silica NPs, (**b**) SS-nPM silica nanocomposites, (**c**) 3F based silica NPs, and (**d**) SS-3F silica nanocomposites.

The integration of spider silk protein with organo- and fluoro-silanes, referred to as the SS-silica nanocomposites is detailed in the Materials Section. The hybrid particles are prepared by addition of the synthetic spider silk into the acid-hydrolyzed silane solutions followed by microwave-induced solubilization of the SS protein then addition of base to induce condensation. The SS-silica nanocomposites were assessed using SEM, AFM, and infrared spectroscopy. In Figure 1, SEM images reveal nanoparticles (NPs) synthesized from the pure silanes (top row) and the SS-silica hybrid particles (bottom row). The pure fluoro (3F) and organo (nPM) silane NPs formed through the acid/base two-step method form highly regular spheres with sizes generally ranging between 350 and 550 nm. These particles resemble typical NPs prepared via Stöber sol-gel methodology; however, the 3F and nPM particles are prepared without any alcohol as co-solvent. The SS-silica nanocomposite particles, Figure 1, bottom row, are notably smaller than the pure silane controls in the top row. These images also suggest that the SS-nPM particles (Figure 1b) are more monodisperse (<50 nm) than the SS-3F particles in Figure 1d where particle sizes are multimodal, with sizes <50 nm and >300 nm clearly observed.

Both SS-nPM and SS-3F particles appear highly aggregated under SEM observation. The morphological differences among the SS-silica nanocomposites may arise from templating effects of the protein on the sol-gel process, apparently favoring formation of smaller NPs. From the SEM data it, is uncertain if the larger particles in the SS-3F synthesis are also hybrid particles, or whether these reflect a competing process of forming pure silane particles. However, we believe the protein and silane to be integrated into composite particles for the SS-3F synthesis based on the following: (1) There was no evidence of protein fibrils in the SEM and AFM images of the SS-3F systems; (2) the 3F to SS molar stoichiometry was 200:1, which is selected to provide sufficient silane (~200 Da) to interact with SS protein (~100 kDa); and (3) the decrease in particle size when the sol-gel process occurred in the presence of SS protein indicated some type of templating or interaction between organic/inorganic components. Differences between nPM and 3F interactions with SS protein in their hydrolyzed states are also possible, with hydrolyzed 3F exhibiting a higher dipole moment than nPM due to the strong electronegativity of the tri-fluoro moiety pendent to the Si atom in 3F. These effects could yield the differences in the 3F-SS and nPM-SS particles observed in the SEM images.

**Figure 2:**
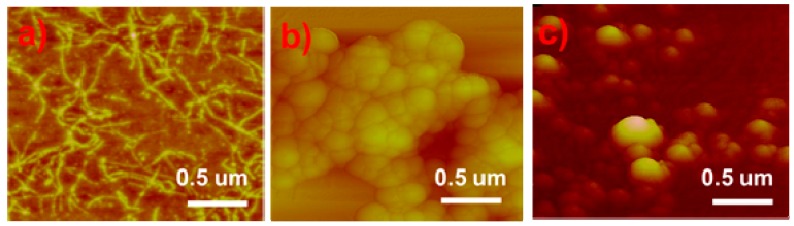
AFM images (**a**) SS proteins, (**b**) SS-nPM silica nanocomposites, and (**c**) SS-3F silica nanocomposites.

AFM was employed as a complementary imaging modality, and in particular, is highly sensitive to analyzing pure SS fibrils, as shown in Figure 2a. No fibrillated proteins were observed upon addition of either silane, as shown in Figure 2b,c. During the microwave synthesis, the SS proteins apparently integrated with the nPM and 3F hydrolyzed monomers and condensation products, thus inhibiting silk protein self-assembly into the characteristics fibrils shown in Figure 2a. The SS-nPM silica nanocomposites and SS-3F silica nanocomposites shown in panels b and c, respectively, reveal the particles to be highly aggregated as observed in the SEM images.

**Figure 3:**
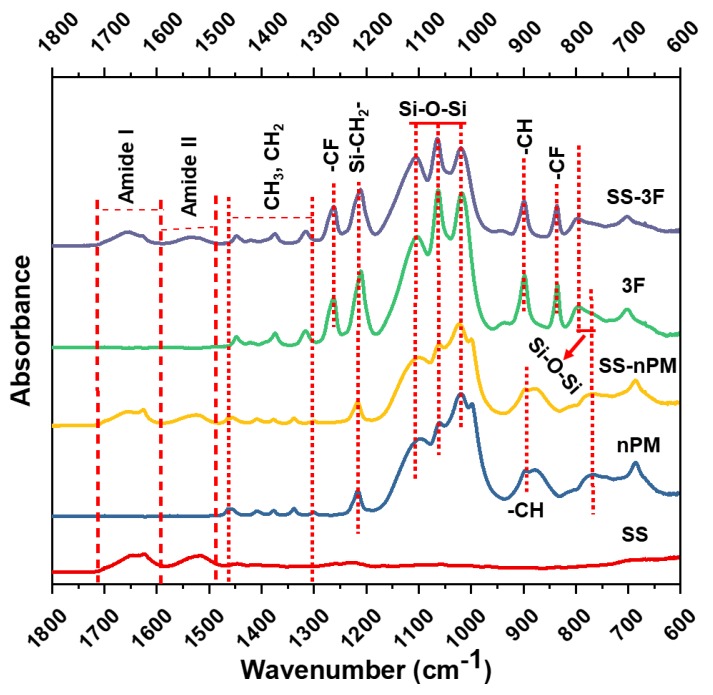
ATR-FTIR spectra of nPM based silica NPs, 3F based silica NPs, SS-nPM based silica nanocomposites (SS-nPM), SS-3F based silica nanocomposites (SS-3F), and SS proteins.

ATR-FTIR spectra (Figure 3) showed some major conformational changes in the amide I zone (1575 cm^−1^ to 1725 cm^−1^) of SS proteins within the SS-nPM silica nanocomposites, and SS-3F silica nanocomposites compare to the SS proteins alone. Figure 3 also includes spectra for the pure nPM and 3F silica NPs. With the amide I and amide II zones, other bending and stretching peaks are also shown in the respected spectra. Spectra representing 3F based silica NPs and SS-3F based silica nanocomposites show the C-F stretching and deformation at 840 cm^−1^, 1210 cm^−1^, and 1260 cm^−1^ [38]. Peaks at 1210 cm^−1^ representing the deformation vibration of Si-CH_2_- exist in both 3F and nPM, causing the increased adsorption intensity at 1210 cm^−1^ in 3F based silica NPs and SS-3F silica nanocomposites as it overlaps with C-F vibration [39]. Strong bands between 1210–1000 cm^−1^ are due to the stretching vibration of Si-O-Si observed in all spectra except the SS spectrum confirm the presence of 3D polymeric network of SiO_2_ [39,40]. Another adsorption band around 800 cm^−1^ is also observed representing Si-O-Si in all the spectra of silica-containing samples [41].

Additionally, the peaks for CH_3_, CH_2_ (1320-1450 cm^−1^), and –CH (900 cm^−1^) were visible in all the spectra except spectrum for SS protein [39]. The presence of Amide I and II in SS-nPM and SS-3F silica nanocomposites along with the other peaks available for nPM and 3F based silica NPs confirm the integration of the SS in the particles observed in SEM and AFM.

**Figure 4:**
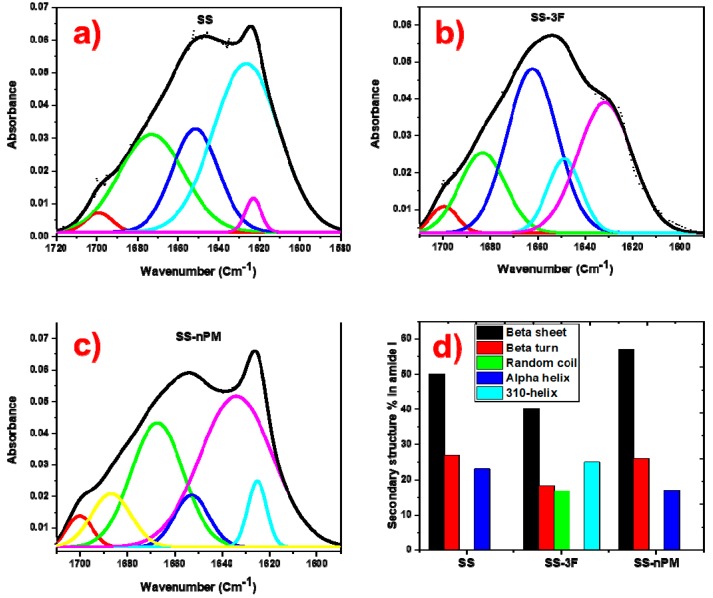
Deconvoluted ATR-FTIR spectra of (**a**) SS proteins, (**b**) SS-3F silica nanocomposites, (**c**) SS-nPM silica nanocomposites, and (**d**) conformational change in secondary structures of BLG.

The spectra representing the nanocomposites and SS protein were deconvoluted in their amide I zone to quantify the conformational changes occurred in the secondary structures due to the interaction of SS protein with nPM and 3F during the formation of nanocomposites. The deconvoluted FTIR spectra of SS, SS-3F, and SS-nPM are shown in Figure 4a–c, respectively. Figure 4d provides a comparison of the secondary structure content in the corresponding SS proteins. In SS-3F silica nanocomposites, β-sheet was reduced by 20%, and β-turn reduced by 41% compared to the spectra reporting these secondary structures in SS proteins alone. In the SS-3F silica nanocomposites two new secondary structures corresponding to a 3_10_-helix appeared and was 25% of the total secondary structures present in the SS-3F nanocomposites.

An opposite trend was observed in the secondary structures in the amide I zone of SS-nPM silica nanocomposites, where both of the primary β structures (sheet and turn) increased. In these nanocomposites, β-sheet increased by 14%, β-turn and α-helix decreased by 4% and 26%, respectively. These patterns of conformational changes in the secondary structures of SS proteins are consistent with conformational changes observed in BLG protein, as reported by Peng et al. [37]. BLG is a relatively low MW (~18.4 kDa), water soluble, globular protein in contrast to the 65–120 kDa hydrophobic SS proteins investigated here. [42] The solubilization of the SS proteins in the presence of the silanes through microwave energy solubilization procedure provides a facile means to synthesis hybrid silane-biomacromolecule nanoparticle–microparticle composites.

## 3. Materials and Methods

3,3,3-trifluoropropyl trimethoxy silane (3F, > 95% purity, MW = 218.3, d = 1.14 g/mL), and n-propyltrimethoxy silane (nPM, > 95% purity, MW = 164.3, d = 0.94 g/mL) were purchased from Gelest, Inc. (Morrisville, PA, USA). 50% (*w*/*w*) NH_4_OH was purchased from Fisher Scientific. Muscovite mica was purchased from SPI supplies for AFM study (West Chester, PA, USA). AFM tips were obtained from TED Pella Inc., Redding, CA (TAP300AL-G-50). Recombinant spider silk protein was obtained from R. Lewis, Utah State University Department of Biology. Two different types of SS protein such as major ampullate silk protein rMaSp1 and major ampullate silk protein rMaSp2 were purified from the milk of transgenic goats through tangential flow filtration, precipitation, and washing, yielding 65–120 kDa proteins. [42]

The hybrid silane/protein composites were prepared by first preparing the silane solutions. 3F (0.4 M) and nPM (0.4 M) were hydrolyzed at pH 3.0 in aqueous media in separate vials. Two SS proteins (rMaSp1and rMaSp2) were mixed with a ratio of 50:50 [1% *w*/*v* or ~0.001 M) and added in 5 mL 3F and nPM media separately after diluting hydrolyzed 3F and nPM solution to 0.02 M which make stoichiometry of 3F/nPM:SS as 200:1. The protein remained as a visible white aggregate in the solution. These mixed solutions were then microwaved (Haier microwave (700 W) for 8 s, followed by a 30 s pause, for 4 times in a tightly closed glass vials to dissolve the SS. Following 4 cycles, the temperature in the vials reached 130 °C and the resulting solutions were clear.

**Figure 5:**
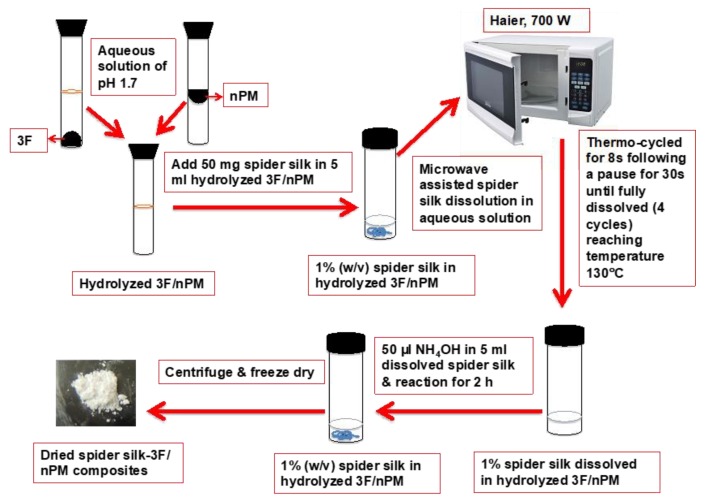
Schematic of Experimental procedure of the spider silk and 3F/nPM nanocomposite materials.

Following microwave-facilitated dissolution of the SS protein, the solutions were cooled to 23 °C using an ice water bath. A 5 mL aliquot of the solution was removed, stirred at 500 rpm, and 50 µL of NH_4_OH was added dropwise to catalyze 3F/nPM condensation. A cloudy sol formed and after 2 h the solution was transferred to 15 mL centrifuge tubes, centrifuged at 4000 rpm to form a pellet. The supernatant was discarded and pellet resuspended in DI water. The centrifugation and wash procedures were repeated two times. The wet pellet was then frozen at −80 °C and lyophilized for 12 h. The dried powder of SS-nPM or SS-3F was collected and stored for analysis. The experimental schematic is shown in Figure 5.

Dried nanocomposite material morphologies were analyzed using AFM and SEM. SEM was performed with a FEI Quanta FEG 650 equipped with an Oxford X-Max energy dispersive X-ray spectroscopy (EDS), (ThermoFisher Scientific, Hillsboro, OR, USA) housed in the Microscopy Core Facility at Utah State University. Samples were imaged with 20 kV accelerating potential without conductive coatings. AFM images were taken using Nanoscope III Bioscope (Digital Instrument, Inc., Camarillo, CA, USA) in tapping mode with Al-coated BS-Tap 300 cantilevers from Budget Sensor, Sofia, Bulgaria. 100 µL samples were drop-cast on freshly cleaved mica surfaces then gently washed with DI water and air-dried before taking AFM images.

Bonding between spider silk protein and both fluorinated and methylated silica were analyzed by FTIR using a Varian 660-IR (Agilent, Santa Clara, CA, USA) with a horizontal single reflection Pike Technologies MIRacle attenuated total reflectance (ATR) unit, fitted with a ZnSe crystal. 100 µL samples were drop cast on the ZnSe crystal of the ATR platform and air-dried before taking the reading. Readings were taken after averaging 20 scans over the range of 600 cm^−1^ to 1800 cm^−1^ with a resolution of 1 cm^−1^. Prior to each reading, a background scan was acquired. The ATR-FTIR spectra were later deconvoluted using OriginPro, OriginLab Corporation (Northampton, MA, USA), to analyze the transformation in the secondary structures of spider silk protein.

## 4. Conclusions

An aqueous sol-gel process combined with microwave-assisted dissolution of hydrophobic synthetic spider silk is demonstrated here to yield silk-silica nanocomposite particles. Sub-micron particles were observed for both silk ORMOSIL (SS-nPM) and silk-F-ORMOSIL (SS-3F) hybrids. Incorporation of SS into the sol-gel process yielded relatively spherical 3F and nPM silk nanocomposites with a greater range of sizes and morphologies, as contrasted with the pure nPM and 3F silica NPs. In the absence of the two hydrophobic silanes, pure SS assembled into fibrillar strands exhibiting strong amide I and II peaks in the ATR-FTIR spectra. Shifts in these peaks in the nanocomposites further confirmed an intimate integration of the SS protein with the silanes in the nanocomposites. However, the influence of the organo- and fluoro-silanes on the SS secondary structures were distinct. Deconvoluted ATR-FTIR spectra showed the increased β structures in SS-nPM silica nanocomposites and decreased β structures in the SS-3F silica nanocomposites. The ability to induce defined secondary structures in the protein-silane hybrid particles may allow for bottom-up design of bioactive particles, surfaces, and monoliths where subsequent epitaxial growth and biomineralization can be tuned for user-defined applications.
